# Primary and Repeated Respiratory Viral Infections Among Infants in Rural Nepal

**DOI:** 10.1093/jpids/piy107

**Published:** 2018-11-12

**Authors:** Jim Boonyaratanakornkit, Janet A Englund, Amalia S Magaret, Yunqi Bu, James M Tielsch, Subarna K Khatry, Joanne Katz, Jane Kuypers, Laxman Shrestha, Steven C LeClerq, Mark C Steinhoff, Helen Y Chu

**Affiliations:** 1 Division of Allergy and Infectious Diseases, University of Washington, Seattle; 3 Department of Laboratory Medicine, University of Washington, Seattle; 4 Department of Biostatistics, University of Washington, Seattle; 2 Department of Pediatrics, Seattle Children’s Hospital, University of Washington, Seattle; 5 Department of Global Health, Milken School of Public Health, George Washington University, Washington, DC; 6 Nepal Nutrition Intervention Project, Sarlahi; 7 Department of International Health, Johns Hopkins University, Baltimore, Maryland; 8 Department of Pediatrics and Child Health, Institute of Medicine, Tribhuvan University, Kathmandu, Nepal; 9 Global Health Center, Cincinnati Children’s Hospital, Ohio

**Keywords:** coronavirus, infants, influenza virus, parainfluenza virus, respiratory viruses, rhinovirus, RSV

## Abstract

**Background:**

Respiratory viruses cause significant morbidity and death in infants; 99% of such deaths occur in resource-limited settings. Risk factors for initial and repeated respiratory viral infections in young infants in resource-limited settings have not been well described.

**Methods:**

From 2011 to 2014, a birth cohort of infants in rural Nepal was enrolled and followed with weekly household-based active surveillance for respiratory symptoms until 6 months of age. Respiratory illness was defined as having any of the following: fever, cough, wheeze, difficulty breathing, and/or a draining ear. We tested nasal swabs of infants with respiratory illness for multiple respiratory viruses by using a reverse transcription polymerase chain reaction assay. The risk of primary and repeated infections with the same virus was evaluated using Poisson regression.

**Results:**

Of 3528 infants, 1726 (49%) had a primary infection, and 419 (12%) had a repeated infection. The incidences of respiratory viral infection in infants were 1816 per 1000 person-years for primary infections and 1204 per 1000 person-years for repeated infection with the same virus. Exposure to other children and male sex were each associated with an increased risk for primary infection (risk ratios, 1.13 [95% confidence interval (CI), 1.06–1.20] and 1.14 [95% CI, 1.02–1.27], respectively), whereas higher maternal education was associated with a decreased risk for both primary and repeated infections (risk ratio, 0.96 [95% CI, 0.95–0.98]). The incidence of subsequent infection did not change when previous infection with the same or another respiratory virus occurred. Illness duration and severity were not significantly different in the infants between the first and second episodes for any respiratory virus tested.

**Conclusions:**

In infants in rural Nepal, repeated respiratory virus infections were frequent, and we found no decrease in illness severity with repeated infections and no evidence of replacement with another virus. Vaccine strategies and public health interventions that provide durable protection in the first 6 months of life could decrease the burden of repeated infections by multiple respiratory viruses, particularly in low-resource countries.

Pneumonia is the leading cause of childhood death globally [1, 2]. Respiratory syncytial virus (RSV), the human parainfluenza viruses (HPIVs), human metapneumovirus (HMPV), influenza, coronavirus (CoV), and human rhinovirus (HRV) are common causes of primary and repeated infections throughout life [3]. By their second birthday, approximately half of all children have been infected at least twice with RSV and one-third with HPIV3 [4, 5]. Repeated progression of respiratory viral infections to lower respiratory tract disease in young children can occur as a result of high-risk exposure, an immature immune system, and physiologic factors including small airways [4].

Previous studies from the United States and Europe have found that RSV is a common cause of repeated infections in children and is associated with substantial healthcare utilization [6–9]. However, limited data from developing countries exist regarding repeated respiratory viral infections in young infants, despite the disproportionate burden of illness in these settings [10]. Furthermore, little is known about which respiratory viruses are most likely to cause repeat infections, whether the risk for a second infection declines after the first infection, and whether repeated infections during infancy lead to long-term sequelae later in life. Filling these knowledge gaps might help us to guide vaccine and therapeutic development and implementation priorities for different respiratory viruses in resource-limited settings, identify populations at higher risk for closer monitoring, and pinpoint potential interventions that lower risk for repeated infections.

The objectives of our study were to characterize the incidence, risk factors, and clinical characteristics of initial and repeated respiratory viral infections in a prospective birth cohort of infants in rural Nepal.

## METHODS

### Patient Population

Data from a randomized placebo-controlled trial of maternal vaccination with an inactivated influenza vaccine in rural Nepal from April 2011 to May 2014 were used in this analysis [11]. Women were enrolled in the second to third trimester of pregnancy and randomly assigned to receive vaccine or placebo, and their infants were followed from birth for 180 days. Gestational age was calculated according to the last menstrual period [11, 12]. Birth weight was measured during a postpartum home visit. Small for gestational age was defined on the basis of INTERGROWTH-21st criteria [13], low birth weight was defined as a birth weight of <2500 g, and preterm birth was defined as birth at <37 completed weeks’ gestation.

Study personnel made weekly home visits to capture daily symptom information by recall from mothers of the previous week. Respiratory illness was defined as having any of the following: fever, cough, wheeze, difficulty breathing, and/or a draining ear; collection of a midnasal swab sample by a field worker took place with respiratory illness weekly episodes. A respiratory viral illness episode was defined as the presence of any respiratory symptoms lasting for at least 1 day and having a nasal swab that tested positive for a respiratory virus. A repeated infection was defined as any respiratory symptom in an infant who also had a positive polymerase chain reaction (PCR) result for the same virus that occurred after at least a 14-day continuous symptom-free period after the initial infection. Weekly nasal swabs were collected as long as symptoms continued to be present. The World Health Organization (WHO) Integrated Management of Childhood Illness disease criteria were used to categorize respiratory disease severity [14, 15]. A severity score of 1 through 4 was assigned on the basis of whether the infant had an upper respiratory tract infection, mild lower respiratory tract infection (LRTI), severe LRTI, or very severe LRTI, respectively. Upper respiratory tract infections were limited to the presence of at least 1 respiratory symptom. Mild LRTIs were limited to cough or difficulty breathing with wheezing. Severe LRTIs included these same symptoms and chest wall indrawing. Very severe LRTIs included lethargy, difficulty feeding, cyanosis, convulsions, and/or vomiting. Medical care was defined as that sought from a physician, nurse, or midwife or at a hospital. Care sought from a village doctor, village pharmacist, or dhami/jhakri (traditional healer) was excluded.

Institutional review board approval for the parent trial was obtained from Johns Hopkins University, Cincinnati Children’s Hospital, Tribhuvan University, and the Nepal Health Research Council with deferral from Seattle Children’s Hospital and the University of Washington. The primary trial is registered under ClinicalTrials.gov identifier NCT01034254.

### Respiratory Viral PCR

Respiratory viruses were detected using a PCR panel for RSV, HMPV, HPIV1 through HPIV4, CoV, HRV, and influenza A and B [16–20]. Specimens in which RSV was detected were analyzed also by quantitative RT-PCR of the matrix gene to determine viral loads and subtyped by RT-PCR of the polymerase gene [21]. The lower limit of detection was 1000 copies/mL [21]. Specimens in which influenza was detected were subtyped by RT-PCR that targeted the hemagglutinin gene [22].

### Statistical Analysis

Analysis was conducted using R (R Project, Vienna, Austria). The denominator for incidence of a primary infection was calculated using days from study enrollment until an observed infection, 6 months of age, the end of the study, or loss to follow-up (including death), whichever came earliest. Incidence of a secondary infection with the same virus was calculated similarly, but timing was started from the end of the primary infection. The beginning of each infection for an infant was defined as the first day with any symptoms within 7 days from when a PCR-positive specimen was collected. If multiple viruses were detected in the same illness episode, the infant was counted as being infected with each virus separately and as having a coinfection. The end of each infection for an infant was defined as the last day with symptoms followed by at least 14 consecutive days without symptoms. For infants who had a second infection, differences in the severity of the first and second infection were tested with the Wilcoxon test. Risk factors for primary and repeated respiratory viral infections were identified by a mixed-effects Poisson regression model with backward selection for final model determination. The variables analyzed included maternal smoking, maternal education, maternal influenza vaccination, presence of an indoor biomass cookstove, number of children in the household, low birth weight, small for gestational age, preterm birth, and male sex. We also examined whether previous infection with a respiratory virus had an effect on the risk for infection with a different virus. Because the number of infants infected with multiple viruses in the course of the study was small, we calculated the relative risk for infection with either of 2 different viruses if the infant was infected with either virus previously. A mixed-effects Poisson model was used to calculate the change in risk of a second infection among pairs of related viruses after a previous infection by either virus. We analyzed whether a previous infection with HRV or influenza, HRV or RSV, HMPV or RSV, and HPIV3 or RSV had an effect on the incidence of subsequent infections with either virus. We decided a priori to limit examination for cross-protection to these 4 pairs of viruses to minimize the chance for false-positive results caused by multiple testing. Results of epidemiologic studies have suggested that previous infection with HRV could decrease the risk of infection with either influenza or RSV, and we sought to test whether this phenomenon was present in our study [23, 24]. We also chose to test for an effect between HMPV, HPIV3, and RSV, because these viruses are phylogenetically related, and a recent epidemiology study found evidence for the development of cross-immunity against different respiratory viruses after infection [25].

## RESULTS

### Study Population

Of the 3646 infants enrolled in the study, 3528 underwent surveillance for a respiratory illness and were included in the analysis. The median number days of surveillance was 174 days (interquartile range, 166–179 days). More than 75000 weekly home visits were conducted, and 4204 nasal swabs were collected.

### Incidence of Primary and Repeated Respiratory Viral Infections

Among all the respiratory viruses for which we tested in symptomatic infants, HRV was the leading cause of both primary and repeated respiratory infections (incidence, 1005 per 1000 person-years and 1543 per 1000 person-years, respectively) (Table 1). Six (1.8%) of 336 infants with primary RSV had a second RSV infection. The incidence of a repeated RSV infection was statistically significantly lower than the incidence of a primary infection (adjusted incidence ratio, 0.17 [95% confidence interval (CI), 0.05–0.57]) (Table 1). We successfully subtyped RSV in 4 of these 6 infants in both illness episodes, and in each case, the RSV subtypes for each pair of episodes matched. No significant difference in mean RSV viral loads between the first and second episodes was observed (4.2 vs 4.8 log_10_ copies/mL, respectively; *P* = .35). The median time between the primary and repeat RSV infections was 21.7 days (range, 15–46 days). For comparison, the median time between the primary and repeat influenza infections was 27.8 days (range, 18–60 days) and for repeat HRV infections was 54.4 days (range, 12–158 days). Five (2.8%) of 177 infants with a primary influenza infection experienced a repeat infection. Four of the five infants were infected in the second episode by an influenza genus that was different than that which caused the first episode. Similar to the results for RSV, we found no significant difference in influenza viral loads between the first and second episodes (mean cycle thresholds, 30.9 vs 31.6, respectively; *P* = .86).

**Table 1.** Incidence of First and Second Respiratory Virus Infections

Infection Type	First Infection			Second Infection ( With Same Virus)			Second vs First Infection	
	No. of Cases	py at Risk	Incidence (per 1000 py)	No. of Cases	py at Risk	Incidence (per 1000 py)	IR (95% CI])	Adjusted IR (95% CI)^a^
RSV	336	1613	208	6	78	77	0.25 (0.04–1.55)	0.17 (0.05–0.57)^b^
HMPV	187	1658	113	1	36	27	0.24 (0.03–1.74)	NC
Influenza	177	1660	107	5	35	142	1.33 (0.55–3.24)	1.16 (0.43–3.16)
HPIV1	67	1688	40	0	11	0	NC	NC
HPIV2	45	1690	27	1	9	112	4.21 (0.58–30.54)	NC
HPIV3	166	1666	100	5	30	169	1.69 (0.69–4.12)	1.29 (0.47–3.49)
HPIV4	72	1685	43	2	13	157	3.68 (0.90–15.01)	4.02 (0.97–16.54)
HRV	1341	1334	1005	406	263	1543	1.20 (0.94–1.53)	1.04 (0.80–1.21)
CoV	283	1637	173	7	56	126	0.73 (0.34–1.54)	0.48 (0.18–1.30)

Abbreviations: CI, confidence interval; CoV, coronavirus; HMPV, human metapneumovirus; HPIV, human parainfluenza virus; HRV, human rhinovirus; IR, incidence ratio; NC, did not converge (for viruses with no or only 1 reinfection); py, person-years; RSV, respiratory syncytial virus.

^a^The adjusted IR was computed in a regression that included the following household factors associated with infection: whether the mother smokes, years of maternal education, birth month from June to September, number of children younger than 5 years, low birth weight, male sex, small for gestational age, and preterm birth. Indoor cookstove was excluded because not having one was rare.

^b^Statistically significant result.

The overall incidence of coinfection was 476 per 1000 person-years. The incidence of coinfections with HRV was 1005 per 1000 person-years; only 29% of infants with a primary HRV infection had a coinfection. In comparison, the proportion of infants with a coinfection during a primary illness episode with RSV was 49%, with HMPV 59%, with influenza 27%, with HPIV1 31%, with HPIV2 53%, with HPIV3 57%, and with HPIV4 58%.

### Seasonality of Respiratory Viral Infections

**Figure 1.**
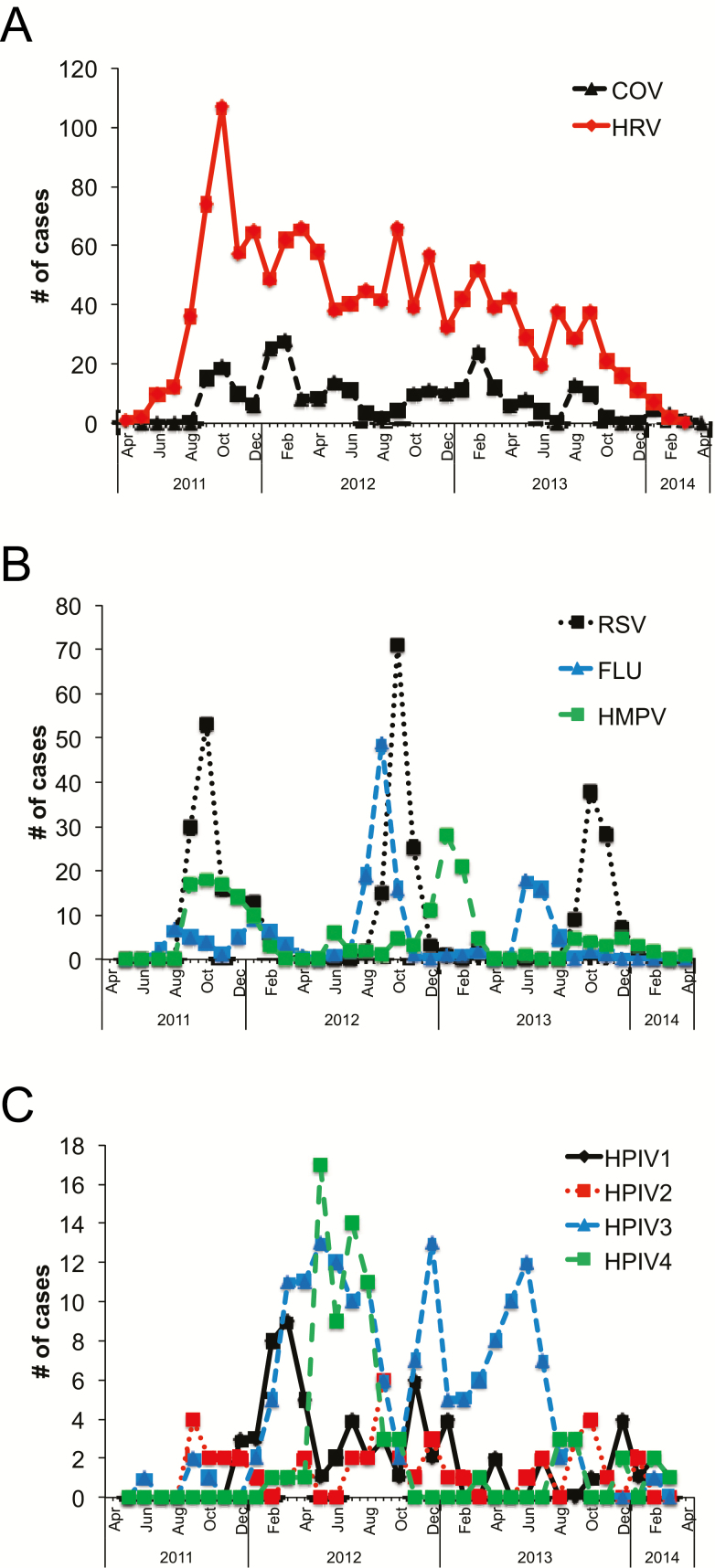
Respiratory viral infections in Nepal. Shown are the numbers of cases of infection caused by coronavirus (COV) and human rhinovirus (HRV) (A), respiratory syncytial virus (RSV), influenza virus (FLU), and human metapneumovirus (HMPV) (B), and parainfluenza viruses types 1 through 4 (HPIV1 through HPIV4) (C) per month from 2011 to 2014 in infants in Nepal. Note that the relatively lower number of infants born and enrolled between April and July 2011 accounts for the lower number of respiratory viruses detected during that time.

HRV circulated year-round and was the most common respiratory viral infection (Figure 1A). The peak times for the other viruses were less consistent between seasons during the study period. In general, HMPV circulated mainly between August and March, and influenza circulated between August and January, although cases were documented nearly year-round (Figure 1B). Cases of HPIV and CoV were seen throughout the year, and a notable spike in HPIV4 cases occurred in the summer/fall of 2012 (Figure 1A and C).

### Characteristics of Infants and Households With a Respiratory Viral Infection

A total of 1341 (38%) infants had a primary respiratory illness with HRV, 334 (9%) with HPIV1, HPIV2, HPIV3, or HPIV4, 336 (10%) with RSV, 283 (8%) with CoV, 187 (5%) with HMPV, and 177 (5%) with influenza (Table 2). The frequency of preterm birth was 447 (13%) of 3528 and of low birth weight was 752 (22%) of 3528 in the overall cohort. The median number of other children younger than 5 years in the household was 1 (range, 0–8), and 1313 (43%) of 3528 households had a household member who smoked. In comparison, for infants with HRV infection, the frequency of preterm birth was 17 (13%) of 1341, of low birth weight was 303 (23%) of 1341, and of household smoking was 506 (44%) of 1341.

**Table 2.** Characteristics of Infants and Households According to Primary Virus Infection

Characteristic	All		RSV		HMPV		Influenza		HRV		HPIV1–HPIV4		CoV	
Infants (n [%])	3528	(100)	336	(10)	187	(5)	177	(5)	1341	(38)	334	(9)	283	(8)
Male (n [%])	1861	(53)	186	(55)	122	(65)	97	(55)	730	(54)	175	(52)	159	(56)
Preterm birth (n [%])^a^	447	(13)	65	(19)	26	(14)	21	(12)	172	(13)	43	(13)	34	(12)
Low birth weight (n [%])^b^	752	(22)	85	(26)	41	(22)	39	(22)	303	(23)	59	(18)	56	(20)
SGA (n [%])^c^	1239	(37)	133	(41)	63	(36)	66	(38)	482	(37)	122	(38)	91	(33)
Born during June–September (n [%])^d^	1538	(44)	248	(74)	128	(68)	74	(42)	585	(44)	101	(30)	123	(43)
Other children <5 years old in the household (mean no. [range])	1	(0–8)	1	(0–8)	1	(0–4)	1	(0–3)	1	(0–5)	1	(0–5)	1	(0–5)
Maternal age (mean [range]) (years)	22	(13–45)	22	(14–41)	23	(15–41)	23	(16–45)	23	(14–41)	22	(15–41)	23	(15–40)
Influenza vaccination in the mother (n [%])	1767	(50)	159	(47)	94	(50)	75	(42)	668	(50)	171	(51)	125	(44)
Maternal education (mean [range]) (years)	5	(0-18)	0	(0-15)	2	(0-15)	2	(0-15)	3	(0-16)	3	(0-15)	3	(0-16)
Indoor biomass cookstove (n [%])	3005	(100)	291	(99)	171	(98)	154	(99)	1136	(99)	293	(100)	242	(100)
Latrine in home (n [%])	1490	(49)	122	(41)	66	(38)	67	(43)	489	(43)	126	(43)	102	(42)
Electricity in the home (n [%])	2723	(90)	252	(87)	156	(90)	136	(88)	1014	(89)	264	(90)	220	(91)
Household smoking (n [%])	1313	(43)	125	(43)	71	(41)	74	(48)	506	(44)	128	(44)	120	(49)

Abbreviations: CoV, coronavirus; HMPV, human metapneumovirus; HPIV, human parainfluenza virus; HRV, human rhinovirus; RSV, respiratory syncytial virus; SGA, small for gestational age.

^a^Preterm was defined as birth at <37 weeks’ gestation.

^b^Low birth weight was defined as a birth weight of <2500 g.

^c^SGA was defined on the basis of INTERGROWTH-21st criteria [13].

^d^June through September corresponds to the monsoon season in Nepal, which precedes the period of peak RSV and influenza circulation.

### Clinical Presentation of Initial and Repeat Respiratory Viral Infections

The most common symptom observed for a first infection with RSV (292 [87%] of 336), HMPV (148 [79%] of 187), HRV (879 [66%] of 1341), HPIV1 through HPIV4 (248 [74%] of 334), and CoV (197 [70%] of 283) (Table 3) was cough. In contrast, the most common symptom for the first infection with influenza was fever (136 [77%] of 177). HRV infection was associated with the highest absolute number (407) of infants who received care from a certified medical provider because of the relatively large number (1341) of HRV infections. However, the proportion of RSV-infected infants who received medical care (152 [45%] of 336; *P* < .001) and developed wheezing (211 [63%] of 336; *P* < .001) or difficulty breathing (192 [57%] of 336; *P* < .001) was higher than that of those who had any other virus.

**Table 3.** Initial Clinical Presentation of Infants With a Respiratory Viral Infection

Infection Type (N)	Clinical Symptom or Characteristic (n [%])^a^					
	Fever	Cough	Wheeze	Difficulty Breathing	Draining Ear	Medical Care Sought
RSV (336)	212 (63)	292 (87)	211 (63)	192 (57)	21 (6)	152 (45)
HMPV (187)	98 (52)	148 (79)	99 (53)	96 (51)	11 (6)	58 (31)
Influenza (177)	136 (77)	99 (56)	71 (40)	67 (38)	8 (5)	64 (36)
HRV (1341)	653 (49)	879 (66)	642 (48)	578 (43)	74 (6)	407 (30)
HPIV1–HPIV4 (334)	228 (68)	248 (74)	179 (54)	152 (46)	16 (5)	119 (36)
CoV (283)	157 (55)	197 (70)	134 (47)	113 (40)	12 (4)	87 (31)
Any virus (3528)	1484 (42)	1863 (53)	1336 (38)	1198 (34)	142 (4)	887 (25)

Abbreviations: CoV, coronavirus; HMPV, human metapneumovirus; HPIV, human parainfluenza virus; HRV, human rhinovirus; RSV, respiratory syncytial virus.

^a^Each percentage is of the total number of cases for each virus.

Among infants with repeated infections, the proportion who had symptoms of fever during the second episode of HRV was higher than the first infection (212 [52%] of 406 vs 175 [43%] of 406, respectively; *P* = .009) (Table 4). Cough was observed more frequently during the second HRV episode (293 [72%] of 406 vs 265 [65%] of 406, respectively; *P* = .03). Wheezing and difficulty breathing occurred in approximately half of the infants who had repeated HRV infections. In addition, approximately half of the infants with repeated HRV infections received medical attention. Of the 207 infants with wheezing during their second HRV infection, 29% (60) had at least one other respiratory viral pathogen codetected in their nasal swab sample. Of the 188 infants with difficulty breathing during their second HRV infection, 26% (49) had at least one other respiratory viral pathogen codetected in their nasal swab sample.

**Figure 2.**
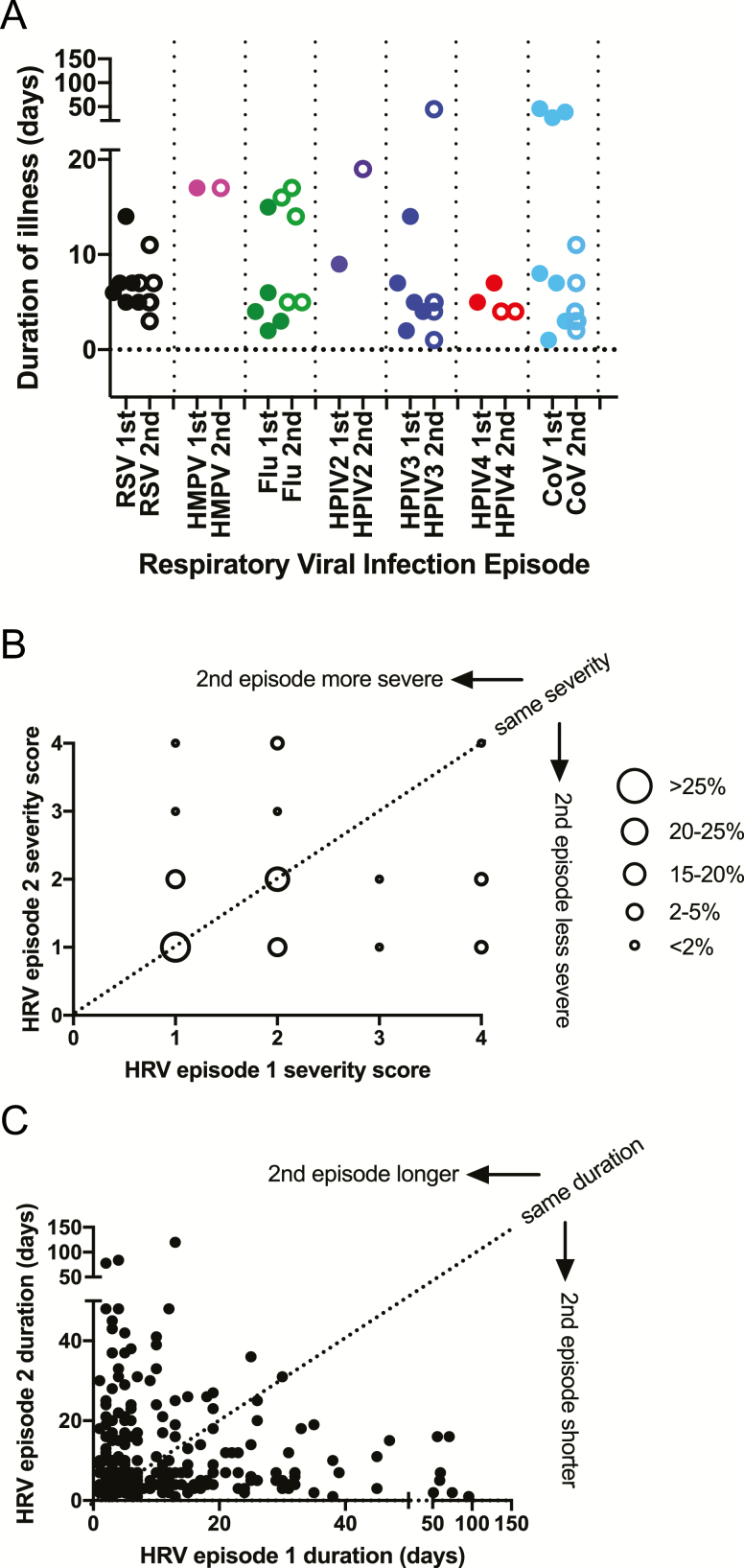
Clinical characteristics of respiratory viral infections in infants with repeated infections. (A) Durations of respiratory illness in infants with repeated infections of respiratory syncytial virus (RSV), human metapneumovirus (HMPV), influenza virus (Flu), human parainfluenza virus (HPIV), human rhinovirus (HRV), and coronavirus (CoV) during the first and second episodes. Each point represents 1 infant. Episodes for which not enough clinical data were available to assign severity are not shown and were not included in the analysis. Closed circles denote the first infection; open circles denote the second infection. (B) Severity for the first and second HRV episodes in infants with repeated infections. The size of each bubble reflects the percentage of infants with repeated HRV infections. Severity scores were based on the World Health Organization Integrated Management of Childhood Illness disease criteria. A severity score of 1 through 4 was assigned on the basis of whether the infant had an upper respiratory tract infection, mild lower respiratory tract infection (LRTI), severe LRTI, or very severe LRTI, respectively. (C) Durations of the first and second HRV episodes in infants with repeated infections. Each point represents 1 infant.

Respiratory disease severity and duration were not significantly different between the first and second episodes for any of the respiratory viruses (Figure 2A and Table 4). The mean severity score for all first infections was 1.6, and the mean score for all second infections was 1.6. The mean duration of illness for all first infections was 7.3 days, and the mean duration for all second infections was 9.6 days. All repeatedly infected infants with severe disease (n = 32) were found to have HRV in the first, second, or both episodes. In addition, all repeatedly infected infants who had symptomatic illness for more than 50 days (n = 10) were infected with HRV. However, a coinfection with a different respiratory viral pathogen was detected during the first episode in 4 of these infants and during the second episode in another 4 infants. Because HRV was the most common etiology of repeated infections by the same virus, we examined the severity and duration of the paired first and second episodes for each infant with HRV (Figure 2B and C). More than half (51%) of the infants with repeated HRV infections experienced the same disease severity in both episodes.

**Table 4.** Clinical Presentation of Infants With Initial and Repeat Respiratory Viral Infections

Infection Type (N)	Episode	Clinical Symptom or Characteristic (n [%])						Severity (Mean [Range])^a^
		Fever	Cough	Wheezing	Difficulty Breathing	Draining Ear	Medical Care Sought	
RSV (6)	First	4 (67)	6 (100)	2 (33)	3 (50)	0	3 (50)	1.2 (1–2)
	Second	2 (33)	4 (67)	4 (67)	2 (33)	0	4 (67)	1.8 (1–2)
HMPV (1)	First	1 (100)	1 (100)	1 (100)	1 (100)	0	0	2
	Second	1 (100)	1 (100)	0 (0)	1 (100)	0	1 (100)	1
Influenza (5)	First	4 (80)	4 (80)	3 (60)	2 (40)	1 (20)	0	1.5 (1–2)
	Second	3 (60)	3 (60)	1 (20)	2 (40)	1 (20)	2 (40)	1.3 (1–2)
HRV (406)	First	175 (43)	265 (65)	208 (51)	192 (47)	22 (5)	102 (25)	1.6 (1–4)
	Second	212 (52)	293 (72)	207 (51)	188 (46)	24 (6)	192 (47)	1.6 (1–4)
HPIV1–HPIV4 (8)	First	4 (50)	6 (75)	5 (63)	2 (25)	0	3 (38)	1.6 (1–2)
	Second	4 (50)	6 (75)	5 (63)	3 (38)	0	5 (63)	1.6 (1–2)
CoV (7)	First	2 (29)	5 (71)	4 (57)	4 (57)	0	0 (57)	1.6 (1–2)
	Second	2 (29)	3 (43)	1 (14)	2 (29)	0	1 (14)	1.1 (1–2)
Any virus (433)	First	190 (44)	287 (66)	223 (52)	204 (47)	23 (5)	111 (26)	1.6 (1–4)
	Second	224 (52)	310 (72)	218 (50)	198 (46)	25 (6)	209 (48)	1.6 (1–4)

Abbreviations: CoV, coronavirus; HMPV, human metapneumovirus; HPIV, human parainfluenza virus; HRV, human rhinovirus; RSV, respiratory syncytial virus.

^a^Severity scores were based on the World Health Organization Integrated Management of Childhood Illness disease criteria. A severity score of 1 through 4 was assigned on the basis of whether the infant had upper respiratory tract infection, mild lower respiratory tract infection (LRTI), severe LRTI, or very severe LRTI, respectively.

### Risk Factors for Respiratory Viral Infections

The presence of other children younger than 5 years in the household (risk ratio [RR], 1.17 [95% CI, 1.11–1.23]), male sex (RR, 1.14 [95% CI, 1.02–1.27]), and lower maternal education (RR, 0.96 [95% CI, 0.95–0.97]) were associated with increased risk for first infection with any respiratory virus (Table 5). Maternal smoking was associated with an increased risk for first infection with any respiratory virus in the bivariate analysis but not in the multivariate analysis. We did not identify any statistically significant risk factors associated with being infected specifically with RSV more than once, but our analyses had very low statistical power to detect such an association (see Supplementary Table 1). Higher maternal education was associated with a decreased risk of repeat infections (RR, 0.95 [95% CI, 0.92–0.98]). No significant effect of maternal influenza vaccination on the overall risk of infection with a respiratory virus in aggregate was found (Table 5).

**Table 5.** Risk Factors for Respiratory Viral Infections

Risk Factor	First Infection With Any Virus				Reinfection With Any Virus			
	Univariable (RR [95% CI])^a^	*P*	Multivariable (RR [95% CI])	*P*	Univariable (RR [95% CI])^a^	*P*	Multivariable (RR [95% CI])	*P*
Maternal smoking	1.48 (1.13–1.95)	.0045	1.26 (0.95–1.68)	.11	1.52 (0.84–2.78)	.17		
Years of maternal education	0.96 (0.95–0.97)^b^	<.0001^b^	0.96 (0.95–0.98)^b^	<.0001^b^	0.95 (0.92–0.98)^b^	.00^b^	0.95 (0.92–0.98)^b^	.00^b^
Indoor cookstove	0.77 (0.33–1.53)	.45			0.47 (0.11–2.07)	.32		
Birth June–September	1.07 (0.97–1.17)	.18			0.83 (0.65–1.05)	.11		
No. of children <5 years old	1.17 (1.11–1.23)^b^	<.0001^b^	1.13 (1.06–1.20)^b^	.0001^b^	1.14 (1.00–1.31)	.051		
Low birth weight	1.02 (0.91–1.15)	.70			0.98 (0.73–1.29)	.83		
Male	1.08 (0.98–1.19)^b^	.10^b^	1.13 (1.01–1.26)^b^	.033^b^	1.19 (0.94–1.50)	.16		
SGA	0.98 (0.89–1.08)	.70			0.81 (0.63–1.04)	.09		
Preterm	1.14 (0.99–1.31)	.070			1.00 (0.71–1.41)	.99		
Received influenza vaccination	0.94 (0.86–1.03)	.20			1.05 (0.83–1.32)	.71		

Abbreviations: CI, confidence interval; NC, does not converge (risk factor was too rare); RR, risk ratio; SGA, small for gestational age.

^a^Adjusted for 3-month period after birth allowing for different risks of repeated infections for infants aged 0 to 3 months than for those aged 3 to 6 months.

^b^Statistically significant result.

We next examined whether previous infection by one virus had an effect on the risk for subsequent infection with a different but related virus. We found no significant change in the incidence of a second infection with either HRV or influenza after a previous infection with either virus (449 second infections; see Supplementary Table 2). We also found no significant change in the incidence of a second infection between HRV and RSV (466 second infections), HMPV and RSV (33 second infections), and HPIV3 and RSV (27 second infections) after a previous infection with either virus.

## DISCUSSION

Using a large prospective birth cohort in rural Nepal with weekly active home-based surveillance for respiratory illness, we characterized the risk factors and clinical characteristics of primary and repeated infections with respiratory viruses among infants younger than six months.

The incidence of RSV infection in infants was similar (208 per 1000 person-years) to that previously described in other studies of respiratory viral infections in infants, including the Seattle Virus Watch program in the 1960s and, more recently, the BIG-LoVE study in Utah, the ORChID study in Australia, and a study in Turku, Finland, that found that the incidence of RSV infection ranged from 167 to 360 per 1000 person-years [6–8, 26]. In a birth cohort in Kenya, the incidence of RSV infection was 487 per 1000 person-years [27]. Despite differences in virus-detection methods, geographic location, and sociodemographic factors, the incidence of RSV infection in children when active case surveillance was carried out was remarkably high and relatively consistent across the studies.

The incidence of initial and repeated HRV infections was higher than that of any other virus tested. Risk factors for HRV infection in infants, especially in the developing world, have not been well described. In prospective birth cohorts in the Netherlands and Australia, the prevalence of HRV infection was higher among infants who attended daycare [28, 29]. In the Australian study, gestational smoking was also associated with significantly increased risk for HRV-C infection. Exposure to smoke in our study in Nepal was high; an indoor biomass cookstove in most households and in 43% of households had a member who smoked and as a result, we might have been unable to detect an association between smoking and the risk for respiratory viral infection. We found that HRV was not detected more frequently in preterm infants or infants born during the monsoon season, unlike RSV, HMPV, and CoV [15, 30]. Male sex, living with other children, and low maternal education were significantly associated with increased risk for infection with any respiratory virus. One possible explanation for the effect we observed for maternal education is that infants born to mothers with higher education have better nutrition or fewer exposures. Although the results are correlative, closer monitoring of these groups and interventions to improve maternal education in the developing world could help reduce the burden of respiratory viral infections.

In this study, the incidence for a second RSV infection was significantly lower than that for the first infection. In a study of 47 households with infants (493 individuals) in Kenya, 155 individuals were infected with RSV once, 22 were infected twice, and two were infected three times [31]. Similar to our findings in Nepal, the study in Kenya also found that repeated RSV infections in infants were caused by the same RSV subtype [32]. Studies in Kenya that used whole-genome next-generation sequencing also found that RSV infections in the same household arose from the introduction of a single virus followed by the accumulation of nucleotide changes during transmission [33, 34]. The lower incidence of a second RSV infection might suggest that infants younger than 6 months can develop immunologic memory after their primary infection and potentially even after vaccination.

Data on the severity of second infections with the same virus in birth cohorts of infants younger than 6 months are limited. We found that the most severe cases of repeated infections with the same virus were caused predominantly by HRV and that the first infection might not blunt the severity of a second infection among infants with repeated HRV infections in the first 6 months of life. Although coinfection with other viruses might contribute to disease severity and duration, our finding that HRV coinfections were less common than infection with the other viruses tested, except for influenza, suggests that HRV alone could account for the severity and duration of symptoms observed during HRV infection. It is possible also that an underlying host factor predisposes to an increased risk for repeat infection and also an increased risk for wheezing and difficulty breathing, because similar proportions of infants in the first and second episodes had these symptoms. Host factors during infancy, including small airways, immunologic immaturity, and waning maternal antibodies in addition to viral factors, including the existence of multiple species for HRV and CoV, contribute to comparable disease severities between the first and second infections [4].

We also examined repeated infections with different respiratory viruses in our study. Results of epidemiologic studies have suggested that previous infection with HRV could decrease the risk of infection with either influenza or RSV [23, 24]. In our analysis, we did not find evidence to support the notion that a previous infection affects the incidence of a subsequent infection with a different virus. This finding is reassuring, because it implies that vaccination against one respiratory virus might not increase the burden of infection from other respiratory viruses. However, this result should be interpreted with caution, because the number of infants sequentially infected with multiple viruses was small, and this analysis was based on calculating the relative risk for being infected with either of 2 different viruses if the infant was infected with either virus previously rather than looking separately for an effect of each virus versus another.

Our study has several limitations. First, rhinorrhea was not used as part of the definition for symptomatic illness. Therefore, the burden of respiratory viral disease might actually have been higher than that observed in this study. Second, our study might have been subject to recall bias, because the mothers were visited weekly and asked to remember if their child was symptomatic. Third, the infants in the study generally did not undergo chest imaging to diagnose their LRTI. We followed the classification system established by the WHO for infants [14]. The WHO classification scheme was used also in another study of RSV that we conducted, and we believe that this is important and relevant, because most vaccine trial end points include the prevention of LRTI [15]. Fourth, it is possible that repeat infections were not truly distinct reinfections, despite a 14-day symptom-free period. In the case of influenza, four of the five infants with a repeat infection had a different influenza genus detected in the second episode than in the first episode, which confirms that each episode was derived from a distinct virus. Although critically ill infants with an LRTI can shed RSV for longer than 3 weeks, previous studies found that the average duration of shedding for RSV was 11.2 days (95% CI, 10.1–12.3 days) and used a period of 14 days to distinguish between episodes of infection [32, 35–37]. The Childhood Origins of Asthma (COAST) study in Wisconsin found that detection of the same virus by molecular methods after 14 days was unusual [38]. Four children in the Philippines were identified recently with homologous RSV subtype B reinfection [39]. Viral sequencing revealed that antigenic site mutations might have contributed to the homologous reinfections observed. Coinfections, particularly with HRV, were also common and could have contributed to disease severity and duration. Because infants coinfected with multiple pathogens were counted separately for each virus, our results therefore describe the clinical presentation, incidence, and risk factors for having an infection with at least the virus specified. Last, time at risk for a respiratory viral infection might depend on when an infant is born because of the differences in seasonality between respiratory viruses. Therefore, we included birth during the monsoon season in our multivariate analysis, and we did not find a statistically significant association with risk for infection with any virus in aggregate.

The results of our analysis of repeated respiratory infections have important implications. RSV infection has been associated with recurrent wheezing, and infants with repeated infections might be predisposed to developing long-term sequelae later in life, including wheezing or asthma, either through an underlying host factor and/or an effect caused by the virus [40]. Information on asthma in the family was not collected in this study, because few people in the setting in which this study was performed had access to regular medical care, and a formal diagnosis of asthma based on diagnostic standards used in allopathic healthcare settings would be uncommon [41]. Future longitudinal studies using spirometry to follow individuals with a history of repeated respiratory viral infections during infancy would be needed to address this issue. Our finding that the incidence of second infection with RSV was lower than the incidence of the first infection with RSV suggests that vaccination of infants could provide some benefit despite their immunologic immaturity. With the exception of influenza vaccine, no vaccine for any of the respiratory viruses detected is currently available [42]. The RSV vaccine field has advanced significantly in the past decade, and several vaccine candidates are undergoing clinical trials. Maternal vaccination to protect neonates from infection by RSV through maternal antibodies is a strategy being studied [43]. In conclusion, our findings reinforce the importance of developing and evaluating new vaccines and preventive interventions directed against multiple different respiratory viruses, particularly in low-resource countries, to protect this vulnerable population.

## Supplementary Material

piy107_suppl_Supplementary_Tables

## Notes

***Acknowledgments. ***We thank all the Nepal Nutrition Intervention Project - Sarlahi (NNIPS) staff and the mothers and infants enrolled in this trial.

***Financial support. ***This work was supported by National Institutes of Health grants T32-AI007044 (to J. B.), T32-AI118690 (to J. B.), and K23-AI103105 (to H. Y. C.), a grant from PATH (to H. Y. C. and J. A. E.), and Bill and Melinda Gates Foundation grant 50274 (to all the authors).

***Potential conflicts of interest. ***H. Y. C. has received research support from GlaxoSmithKline, Novavax, and Sanofi Pasteur; J. A. E. has received research support from Gilead, Chimerix, GlaxoSmithKline, and Pfizer and served as a consultant for Sanofi Pasteur and Gilead; and M. C. S. serves on the board of the Novartis Vaccine Institute for Global Health. All other authors: No reported conflicts of interest. All authors have submitted the ICMJE Form for Disclosure of Potential Conflicts of Interest. Conflicts that the editors consider relevant to the content of the manuscript have been disclosed.
