# Supplementary material for: Primary and Repeated Respiratory Viral Infections Among Infants in Rural Nepal
Source: J Pediatric Infect Dis Soc. 2018 Nov 12;9(1):21–9. doi: 10.1093/jpids/piy107 (PMC7317152; doi:10.1093/jpids/piy107)
Supplement: Supplementary file 1 [file piy107_suppl_supplementary_tables.docx]

Supplementary Table 1. Risk factors for RSV re-infection

	Univariable*	
	Risk ratio (95% CI)	*P* value
Mom smokes	NC	-
Years of maternal education	0.92 (0.67, 1.27)	0.61
Indoor cookstove	NC	-
Birth June to September	1.54 (0.03, 69.6)	0.82
Number children under age 5	1.81 (0.62, 5.28)	0.28
Low birth weight	1.86 (0.11, 32.65)	0.67
Male infant	0.20 (0.01, 3.64)	0.27
Small for gestational age	1.76 (0.11, 28.26)	0.69
Preterm	3.96 (0.24, 65.62)	0.34

NC = does not converge since risk factor was too rare

*Adjusted for 3-month period following birth, a binary measure allowing different risks of repeated infections for 0-3 versus 3-6 months of life

Supplementary Table 2. Change in incidence for a second infection with a different virus

1st infection	Relative risk for second infection with either virus (CI)	Incidence of first infection per 1000 py	Incidence of second infection per 1000 py	*P*
HRV or FLU	1.24 (0.98 - 1.55)	1073	1654	0.070
HRV or RSV	1.09 (0.89 - 1.34)	1119	1627	0.40
RSV or HMPV	1.05 (0.73 - 1.49)	305	319	0.80
RSV or HPIV3	0.87 (0.59 - 1.28)	304	264	0.48

Abbreviations: py (person-years)
